# Modification of chrysanthemum odour and taste with chrysanthemol synthase induces strong dual resistance against cotton aphids

**DOI:** 10.1111/pbi.12885

**Published:** 2018-02-09

**Authors:** Hao Hu, Jinjin Li, Thierry Delatte, Jacques Vervoort, Liping Gao, Francel Verstappen, Wei Xiong, Jianping Gan, Maarten A. Jongsma, Caiyun Wang

**Affiliations:** ^1^ Key Laboratory of Horticultural Plant Biology MOE Huazhong Agricultural University Wuhan China; ^2^ BU Bioscience Wageningen University and Research Wageningen The Netherlands; ^3^ Laboratory of Plant Physiology Wageningen University and Research Wageningen The Netherlands; ^4^ Laboratory of Biochemistry Wageningen University and Research Wageningen The Netherlands; ^5^ Hubei Collaborative Innovation Center of Dabie Mountains Huanggang Normal University Huanggang China

**Keywords:** chrysanthemol synthase, chrysanthemum, aphid resistance, terpene volatile, glycoside, double bioactivity

## Abstract

Aphids are pests of chrysanthemum that employ plant volatiles to select host plants and ingest cell contents to probe host quality before engaging in prolonged feeding and reproduction. Changes in volatile and nonvolatile metabolite profiles can disrupt aphid–plant interactions and provide new methods of pest control. Chrysanthemol synthase (CHS) from *Tanacetum cinerariifolium* represents the first committed step in the biosynthesis of pyrethrin ester insecticides, but no biological role for the chrysanthemol product alone has yet been documented. In this study, the *TcCHS* gene was over‐expressed in *Chrysanthemum morifolium* and resulted in both the emission of volatile chrysanthemol (ca. 47 pmol/h/gFW) and accumulation of a chrysanthemol glycoside derivative, identified by NMR as chrysanthemyl‐6‐O‐malonyl‐β‐D‐glucopyranoside (ca. 1.1 mM), with no detrimental phenotypic effects. Dual‐choice assays separately assaying these compounds in pure form and as part of the headspace and extract demonstrated independent bioactivity of both components against the cotton aphid (*Aphis gossypii*). Performance assays showed that the *TcCHS* plants significantly reduced aphid reproduction, consistent with disturbance of aphid probing activities on these plants as revealed by electropenetrogram (EPG) studies. In open‐field trials, aphid population development was very strongly impaired demonstrating the robustness and high impact of the trait. The results suggest that expression of the *TcCHS* gene induces a dual defence system, with both repellence by chrysanthemol odour and deterrence by its nonvolatile glycoside, introducing a promising new option for engineering aphid control into plants.

## Introduction

Aphids (Aphididae) are abundant and destructive agricultural pests causing serious economic damage to cultivated plants in the form of stunting, sooty mould and transmitted phytopathogenic viruses (Goggin, 2007). Chrysanthemum (*Chrysanthemum morifolium*) is an important ornamental plant which is particularly susceptible to aphid infestation. Chemical insecticides have been the most common strategy to control these pests, but their widespread application is costly, leads to the development of insecticide‐resistance and may have detrimental effects on the environment and human health (Dedryver *et al*., 2010).

Toxic or antinutritional plant proteins were an early lead to engineering aphid resistance into plants. Proteinase inhibitors (Rahbé *et al*., 2003; Stoger *et al*., 1999; Valizadeh *et al*., 2013) and plant lectins (Michiels *et al*., 2010) yielded significant but mild effects on aphid survival, reproduction, growth or mortality. More recently, plant‐derived secondary metabolites, such as terpenoids (Aharoni *et al*., 2003), alkaloids (Kim *et al*., 2011), hydroxamic acids (Hansen, 2006), green leaf volatiles (GLVs) (Vancanneyt *et al*., 2001) and methyl salicylate (Zhu and Park, 2005), were shown to be effective to various extents against aphids. The volatile terpenoids are the largest and most diverse compound family, and particularly relevant in aphid‐plant interactions, as aphids rely heavily on olfaction for host plant localization or avoiding unsuitable plants (Pickett *et al*., 2013).

In response to herbivory, plants emit blends of volatiles, usually dominated by mono‐ and sesquiterpenes that may operate both in direct defence by repelling aphids and in indirect defence by attracting natural enemies such as parasitoid wasps and predators such as ladybird beetles (Clavijo *et al*., 2012; Lange and Ahkami, 2013). For instance, the monoterpenes citral, myrcene, geraniol, linalool, β‐citronellol and pulegyl alcohol have been shown to induce significant aphid repellence (Gutiérrez *et al*., 1997; Halbert *et al*., 2009; Ricci *et al*., 2006), and the sesquiterpenes farnesol, (*E*)‐β‐farnesene, bisabolene, p‐benzoquinone perezone and guaiol to inhibit aphid feeding activity and settling or attract lacewings and parasitic wasps that attack aphid colonies (Beale *et al*., 2006; Burgueno‐Tapia *et al*., 2008; Dudareva and Pichersky, 2008; Gutiérrez *et al*., 1997; Liu *et al*., 2013; Yu *et al*., 2012). Enhanced emissions of terpenes may, therefore, lead to improved plant defence against aphids and avoid the environmental costs of chemical pest control (Dudareva and Pichersky, 2008).

Notable reports in enhancing direct and indirect plant defences to aphids by modulating terpene volatiles in plants via metabolic engineering, involved S‐linalool in Arabidopsis (Aharoni *et al*., 2003), (*E*)‐β‐farnesene in Arabidopsis and tobacco (Beale *et al*., 2006; Yu *et al*., 2012) and diterpene cembratrieneol in tobacco (Wang *et al*., 2001). Interestingly, the expression of terpenoids often also leads, by the action of endogenous enzymes such as cytochrome P450 enzymes, dehydrogenases and glycosyl transferases, to an array of nonvolatile glycosidic derivatives which are storage forms of terpenoids (Dudareva and Pichersky, 2008). These stored glycosides may release antibiotic or kairomone terpene volatiles upon contact with glycosidases or may be bioactive themselves. Both types of terpenoids may fulfil independent biological roles in plant resistance to insects (Aharoni *et al*., 2003; Pankoke *et al*., 2010; Yang *et al*., 2013). For example, linalool emitted by the transgenic *FaNES1* chrysanthemum attracted western flower thrips (WFT), but the linalool glycosides acted as deterrents (Yang *et al*., 2013). The green leaf volatile (Z)‐3‐hexenol from infested plants is a kairomone for parasitoid wasps, but can also be absorbed by neighbour plants and transformed to a glycoside that decreases the survival rate of cutworms (Sugimoto *et al*., 2014). However, until now, there is no report of a terpenoid volatile and glycoside derivative that yield a dual line of defence against the same organism.

Chrysanthemol synthase (CHS) is the first key enzyme in the biosynthesis of pyrethrins, a widely used plant‐derived biopesticide in pyrethrum (*Tanacetum cinerariifolium*) (Matsuda, 2012). It catalyses the formation of chrysanthemyl diphosphate (CPP) from two molecules of dimethylallyl diphosphate (DMAPP) (Rivera *et al*., 2001), and also the next step of removing the diphosphate to form chrysanthemyl alcohol (chrysanthemol, COH) (Yang *et al*., 2014). *TcCHS* evolved from an ancestral farnesyl diphosphate synthase (FDS) (Hemmerlin *et al*., 2003) and similar genes have been also found in other Asteraceae species, including *chrysanthemum morifolium*, which do not produce pyrethrins. Chrysanthemol was first isolated from *Artemesia ludoviciana* almost forty years ago (Alexander and Epstein, 1975). So far, it has been found as a volatile of *Lippia* (Oliveira *et al*., 2006), *Artemisia* (Costa *et al*., 2009), *Santolina* (Gnavi *et al*., 2010) and *Anthemis* (Pavlovi *et al*., 2010) species. However, a biological role for CHS and chrysanthemol beyond the synthesis of pyrethrins has not been established as yet (Liu *et al*., 2012). In pyrethrum, chrysanthemol has been demonstrated to be produced in trichomes and further modified to chrysanthemic acid before being exported out of the trichomes to synthesize pyrethrins (Ramirez *et al*., 2012).

In chrysanthemum ‘1581’, the expression level of an endogenous homologous *CHS* gene was found to be very low (data not shown). The plants did not produce detectable levels of chrysanthemol and were highly susceptible to aphids. The aim of this study was to test whether overexpression of the *TcCHS* gene in chrysanthemum could produce chrysanthemol or any derivatives and improve defence against aphids. For this, we introduced the *TcCHS* gene from pyrethrum into chrysanthemum and characterized the induced volatiles and nonvolatiles, and their effects on aphid behaviour and development in a series of behavioural assays and field experiments.

## Results

### Generation of *TcCHS* chrysanthemum and characterization of transcript levels

**Figure 1:**
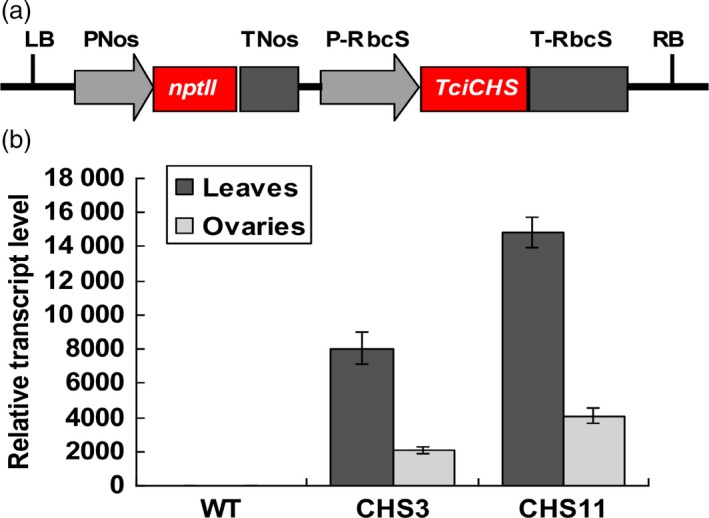
Expression analysis of chrysanthemum plant lines transformed with *TcCHS*. (a) Schematic diagram of the *RbcS* promoter*::TcCHS* gene expression cassettes in the pBINPLUS plasmid. (b) qRT‐PCR analysis of *TcCHS* expression levels in leaves and ovaries of wild type and transgenic chrysanthemum lines relative to *Actin*. The expression was normalized to the *Actin* gene, and gene expression values were averaged across three independent biological replicates, with each sample amplified in triplicate. Error bars represent standard errors (±SE). Expression in wild type was set at 1.

An aphid‐sensitive cultivar chrysanthemum ‘1581’ was transformed with the chrysanthemol synthase gene from pyrethrum, *TcCHS* (JX913537), under a strong rubisco small subunit promoter (Figure 1a). Three‐month‐old cuttings of two T_0_ transgenic lines, line CHS3 and CHS11, were analysed for expression levels of *TcCHS* using quantitative RT‐PCR. The transcript levels of *TcCHS* of two transgenic lines were 8155 and 14802 times higher in leaves and 484 and 1048 times higher in ovaries relative to the household gene *Actin* (Figure 1b).

### Effects of *TcCHS* overexpression on plant phenotype

Overexpression of monoterpene synthases sometimes leads to pleiotropic negative phenotypic effects, such as slowed or stunted plant growth, reduced flower fertility, light green leaves and smaller fruits (Davidovich‐Rikanati *et al*., 2007; Tang *et al*., 2012). The effects on plant development of *TcCHS* expression were therefore measured. No significant differences between wild type and transgenic plants were observed for plant height, dry weight, leaf size, and chlorophyll and carotenoid contents, suggesting that overexpression of the *TcCHS* gene did not negatively affect the plant phenotype (Table 1)

**Table 1:** Plant mean leaf area, chlorophyll and carotenoid contents and dry weight of transgenic and wild type plants

Lines	Leaf area (cm^2^)	Chlorophyll (mg/g FW)	Carotenoids (mg/g FW)	Dry weight (g)
Wild type	11.23 ± 0.07a	1.05 ± 0.05a	0.22 ± 0.01a	0.42 ± 0.02a
CHS3	10.33 ± 0.90a	0.81 ± 0.08a	0.19 ± 0.01a	0.34 ± 0.04a
CHS11	9.56 ± 1.33a	1.01 ± 0.06a	0.22 ± 0.01a	0.39 ± 0.03a

Values are the mean (±SE) of three biological replicates, and different letters within a column indicate significant differences (ANOVA followed by Duncan's multiple range test. *P *<* *0.05).

### Effects of *TcCHS* overexpression on headspace emission from intact plants

**Figure 2:**
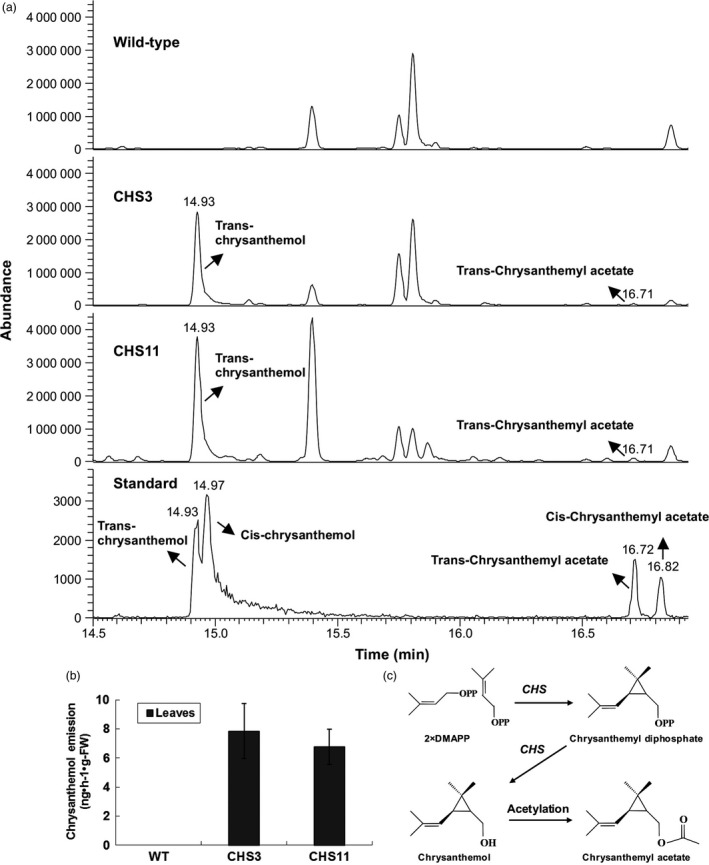
Analysis by GC‐MS of volatile compounds of chrysanthemum plants. (a) Typical GC‐MS chromatograms showing the volatiles by dynamic headspace trapping of intact wild type chrysanthemum plant, transgenic *TcCHS* chrysanthemum plant and an authentic standard of chrysanthemol at RT 14.5–17 min. (b) Chrysanthemol emission of intact wild type and transgenic chrysanthemum lines. Error bars indicate SE from three biological replicates. (c) The pathway of chrysanthemol and chrysanthemyl acetate biosynthesis.

To determine the product of *TcCHS* overexpression, volatiles collected from the headspace of intact plants were analysed by GC‐MS. All differential volatiles were analysed by untargeted metabolomics, comparing all mass signals within compound clusters between different plant lines. In total, 10136 detected mass signals were found to group into 630 clusters. Among these, five clusters showed at least fivefold intensity differences (*P *<* *0.05) between transgenic lines and wild type plants. More clusters were found to be significantly increased (4) than decreased (1) in both transgenic lines, but most of these signals were very low in intensity (Table S1). Only two new compounds were found in transgenic plants. One dominant new peak eluting at 14.92 min was identified as trans‐chrysanthemol (COH) by matching its retention time and mass spectrum to the authentic standard of trans‐chrysanthemol (Figure 2a). It was 7515‐ to 8868‐fold higher compared to background noise in wild type plants. The emission of transgenic lines CHS3 and CHS11 was quantified in leaves at 7.84 and 6.75 ng/h/g fresh weight, respectively (51 and 44 pmol/h/gFW, Figure 2b). Besides trans‐chrysanthemol, the other new peak at 16.71 min was identified as trans‐chrysanthemyl acetate, the acetylation product of trans‐chrysanthemol, by matching it to the authentic standard. The peak intensity was much less high above the background noise in wild type plants (158‐ to 192‐fold) (Figure 2a) (Table S1).

### Effects of *TcCHS* overexpression on the accumulation of nonvolatile compounds

As monoterpene alcohols can be conjugated to glycosyl compounds and stored in the vacuole, the nonvolatile metabolites from leaves were analysed by LC‐MS, and all differential nonvolatiles were analysed by untargeted metabolomics.

**Figure 3:**
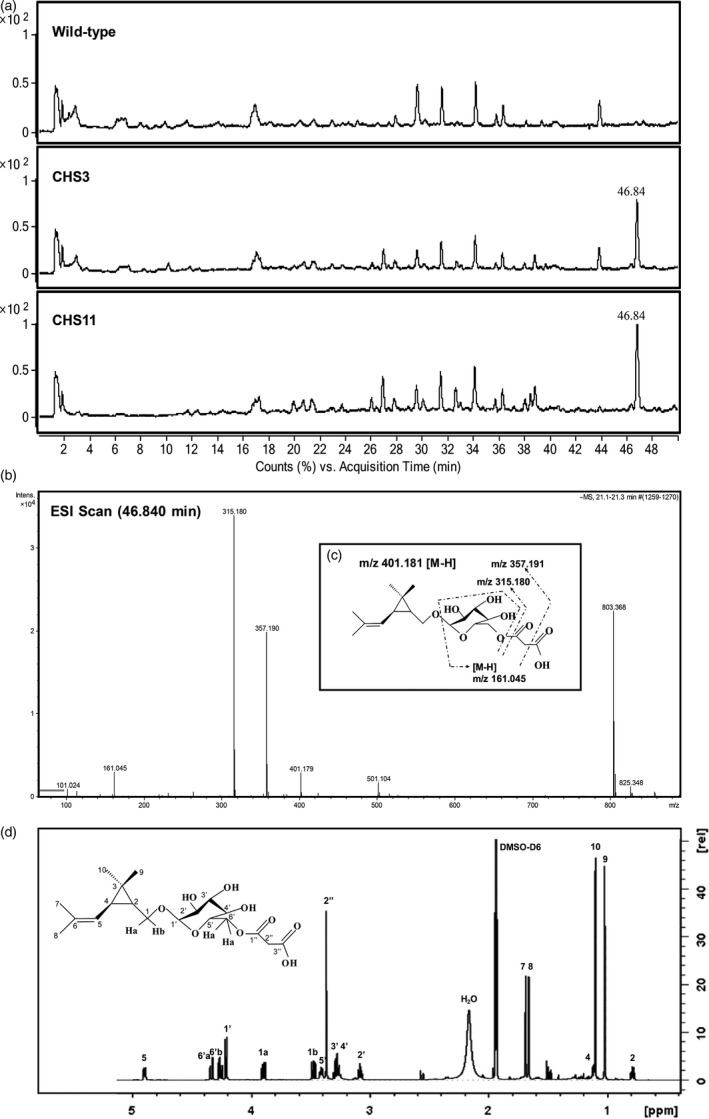
Analysis of nonvolatile compounds of chrysanthemum plants. (a) Negative mode LC‐Q‐TOF‐MS chromatograms of aqueous–methanol extract of leaves of wild type chrysanthemum plant and transgenic *TcCHS* chrysanthemum plant. The compound eluting at 46.84 min uniquely found in the *TcCHS* overexpressing chrysanthemum plants. (b) The MS spectrum of the 46.84 min compound. (c) The spectrum of collision‐induced fragmentation of mass 401.179 eluting at 46.84 min, which was identified by NMR as chrysanthemyl‐6‐O‐malonyl‐β‐D‐glucopyranoside. (d) 600 MHz ^1^ HNMR spectrum of chrysanthemyl‐6‐O‐malonyl‐β‐D‐glucopyranoside.

In total, 811 detected mass signals were found to group into 88 clusters. Among these, 16 clusters showed at least fivefold intensity difference (*P *<* *0.05) when comparing both transgenic lines to wild type plants. More clusters were found to be significantly increased (12) than decreased (4) in both transgenic lines, but most of these signals were very low in intensity (Table S2). Only one new cluster was found only in transgenic plants (mass 803.368), eluting at a retention time of 46.84 min with a peak 214‐ to 256‐fold higher than background noise in wild type plants (Figure 3a and b) (Table S2). Based on its mass spectrum, this new product in *TcCHS* plants was putatively identified as a type of β‐D‐glycoside of chrysanthemol (Figure 3c) and could be hydrolysed by β‐glycosidase (Figure S1).

In order to unambiguously identify the structure of this glycoside, the molecule was subsequently purified from the transgenic plants for NMR analysis using preparative LC‐MS. The mass of 803.368 appeared to be a [2M‐H]^−^ adduct of 401.181. Starting with 10 g fresh leaves, we recovered 562 μg essentially pure compound in 200 μL acetonitrile (13% recovery). Based on one‐ and two‐dimensional NMR data (Figure 3d and Table S3 for the ^13^C NMR and ^1^H NMR data), the structure of the molecule was identified as chrysanthemyl‐6‐O‐malonyl‐β‐D‐glucopyranoside. The content in transgenic line CHS11 chrysanthemum leaves was quantified at 434 μg/g fresh weight (1.08 mM). Chrysanthemol is likely glycosylated and malonylated in one or two steps by endogenous enzymes to generate this compound.

### Effects of *TcCHS* overexpression on aphid performance

**Figure 4:**
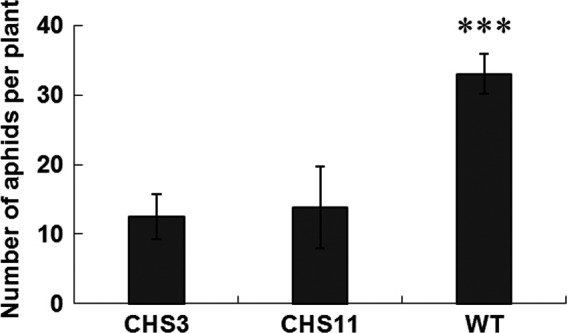
No‐choice whole plant bioassay on transgenic chrysanthemum and wild type plants. Ten neonate larvae were introduced in each clip cage on each plant, and aphids number were measured after 12 days. Wild type plants served as control. Error bars indicate mean ± SE (*n *=* *6). (ANOVA followed by Duncan's multiple range test based on difference of the aphids number. ***: *P *<* *0.001). WT, wild type.

To investigate the effects of overexpression of *TcCHS* on aphid performance, aphids were inoculated on transgenic and wild type plants and the population development was monitored. After a 12‐day period, the total population of aphids on wild type plants was 33/plant, which was 2.4–2.6 times more than those on transgenic plants (Figure 4). This prompted us to further investigate the role of the *TcCHS*‐induced plant volatile and nonvolatile components on aphid behaviour.

### Effects of chrysanthemol on aphid choice and alarm behaviour

**Figure 5:**
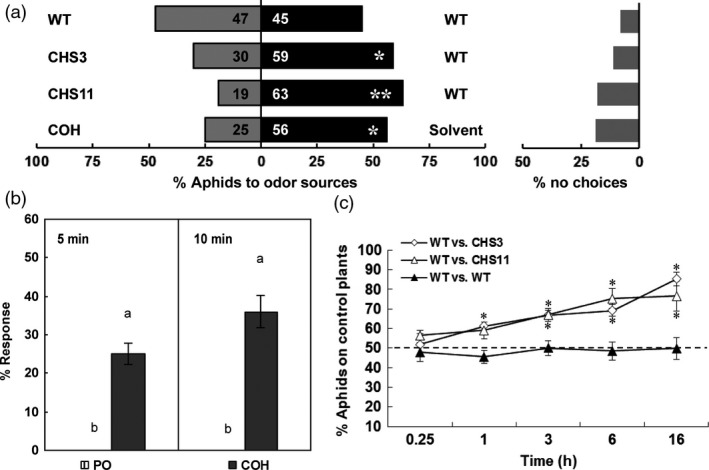
Dual‐choice and alarm behaviour assay of aphids. (a) Olfactory preference of aphids in dual‐choice assay for wild type and transgenic chrysanthemum leaves and 10 μL 10% (v/v) COH standard dissolved in paraffin oil vs 10 μL paraffin oil as positive control. Each bar represents the percentage of aphids that made a choice within 10 min after release (*n *=* *10). The number on the bar represent number of aphids in total. The percentage of aphids did not make a choice (% no choice) indicated on the right. (b) Alarm response of settled aphids to synthetic chrysanthemol in paraffin oil and a paraffin oil control. The percentage of aphids moving after 5 min and 10 min was recorded. Values are the mean ± SE (*n *=* *20). (ANOVA followed by Duncan's multiple range test based on difference of the aphids number.). Columns with different letters indicate responses that are significantly different (*P *<* *0.01). PO, paraffin oil; COH, chrysanthemol. (c) Dual‐choice assay of aphids choosing between wild type and transgenic chrysanthemum plants connected by a paper bridge. The number of aphids on either plant was recorded at 0.25, 1, 3, 6 and 16 h after release. Wild type plants were used as control. Error bars indicate mean ± SE (*n *=* *6). (two‐tailed Wilcoxon signed rank test based on difference of the choices. *: *P *<* *0.05; **: *P *<* *0.01). WT, wild type.

To test the possible role of chrysanthemol on aphid repellence, aphid preference for leaf discs was tested in a dual‐choice assay involving only olfactory cues. Individual aphids were placed on an upright wire between two leaf discs, and their choices were recorded. Almost 80% of aphids chose within 5 min, with 59%–63% repelled by transgenic leaves (*P *<* *0.05) (Figure 5a). Aphids were similarly repelled by a positive control of 10% chrysanthemol in paraffin oil.

As *TcCHS* chrysanthemum was strongly repellent for aphids, an alarm response assay of settled feeding aphids was carried out using a quantity of chrysanthemol roughly corresponding to the emission of chrysanthemol by a whole plant in one hour. The number of settled aphids that started moving 5 and 10 min after exposure to 500 ng chrysanthemol in the headspace was recorded. We found that the response to chrysanthemol in paraffin oil, both after 5 min (25% moving) and 10 min (36% moving) exposure, was significantly higher than to paraffin oil controls (0% moving) (Figure 5b).

Further dual‐choice assays using whole plants and over a longer period established that the repellent effects were independent of leaf disc preparation and were sustained over longer time periods. Aphids were presented with young transgenic and wild type plants by placing them on a paper bridge between two plants. 1 h after aphid release, 59%–61% of the aphids preferred the wild type plants, and the number of aphids on the transgenic plants significant decreased further over time, with 76%–85% of all aphids on the control plants after 16 h (*P *<* *0.05) (Figure 5c). These bioassays strongly indicate that chrysanthemol produced by *TcCHS* overexpression in plants acted as a lasting repellent of aphids.

### Effects of chrysanthemol glycoside induced by *TcCHS* overexpression on aphid deterrence

**Figure 6:**
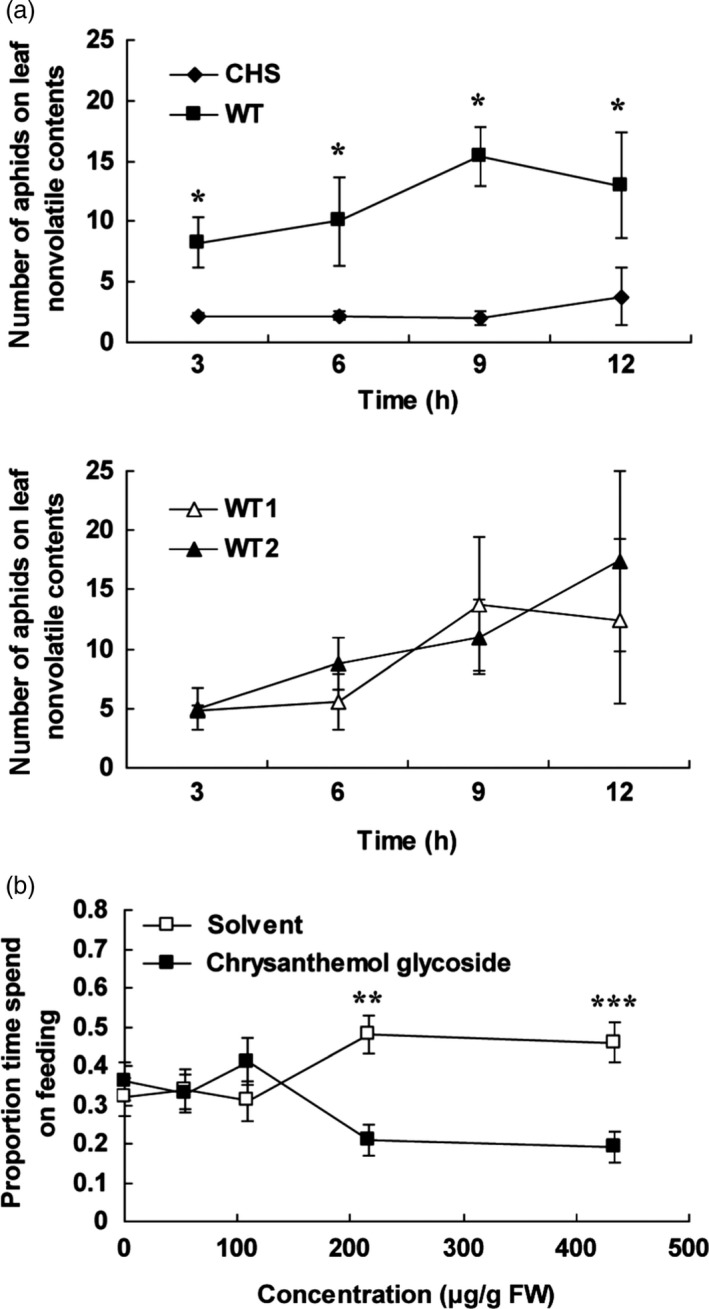
Dual‐choice assay of aphids with nonvolatile contents from plants and pure chrysanthemol glycoside. (a) Aphid preference over time in dual‐choice assay with nonvolatile contents from wild type versus transgenic chrysanthemum leaves and wild type versus wild type. The aphid behaviour was recorded every 15 min for a 12‐h period. The average number of aphids observed underneath either droplets with plant nonvolatile contents was recorded every 15 min. Averages of five replications of two dual‐choice plates with ten aphids in each plate are given per 3‐h time interval. WT, wild type. (b) The ratio of feeding time to total trial duration in dual‐choice assay with a series of dilutions of pure chrysanthemol glycoside and solvent control. Error bars indicate mean ± SE. *n *=* *56. (two‐tailed Wilcoxon signed rank test based on difference of the choices. *: *P *<* *0.05; **: *P *<* *0.01; ***: *P *<* *0.001).

To test whether the chrysanthemol glycoside also has effects on aphid deterrence, a dual‐choice preference assay of aphids on nonvolatiles from *TcCHS* transgenic and wild type leaves were carried out. The plant nonvolatile contents were prepared by evaporating the methanol extract and redissolving it in a water sucrose solution. The chemical composition and quantity of the water‐dissolved plant nonvolatile contents (rechecked by LC‐MS) were comparable to that of the original methanol extracts, and the only major chemical difference between transgenic and wild type leaves was the chrysanthemyl‐6‐O‐malonyl‐β‐D‐glucopyranoside. GC‐MS analysis of the extract confirmed the absence of free volatile chrysanthemol in the extract (data not shown). During the first 12 h, aphids were found in 4–7 times larger numbers on control extracts (Figure 6a).

In order to directly prove that the deterrence activity was related to chrysanthemyl‐6‐O‐malonyl‐β‐D‐glucopyranoside, the pure compound was tested in dual‐choice assays at the concentration of transgenic line CHS11 and four dilutions. Chrysanthemol glycoside at a concentration of 217–434 μg/mL (0.54–1.08 mM) induced a significant deterrence of aphids of roughly a factor 2.5 (Figure 6b). Lower concentrations were not effective. This demonstrates that chrysanthemyl‐6‐O‐malonyl‐β‐D‐glucopyranoside can explain the observed deterrence induced by extracts and likely deters the aphids during test probing of mesophyll, cortex and phloem cells.

### Effects of *TcCHS* overexpression on aphid probing behaviour

Both the volatiles and nonvolatiles of *TcCHS* chrysanthemum induced strong repellence and deterrence of aphids. To find out how this was reflected in the aphid feeding behaviour, we recorded electrical penetration graphs (EPG) of individual aphids on transgenic and wild type plants for 8 h and assessed how often they probed and what stylet activities they performed (Table 2). On both transgenic plants, the total no‐probing (pattern np) time was 0.6–1.5 h longer, and the frequency of stylet penetration of the epidermis and mesophyll (pattern C) was two times more compared to wild type plants. Importantly, the total time that aphids ingested phloem (E2) was 2.5–3 h less on transgenic plants effectively causing a 3–5 times lower food intake. All these elements demonstrated that host plant acceptance was significantly disturbed on transgenic plants.

**Table 2:** EPG parameters of *Aphis gossypii* during an 8‐h recording on transgenic and wild type plants

EPG parameters	CHS3 (*n *=* *12)	CHS11 (*n *=* *12)	Wild type (*n *=* *12)
Duration of np (h)	2.01 ± 0.67**	1.13 ± 0.17*	0.55 ± 0.16
Duration of C (h)	3.25 ± 0.41	4.40 ± 0.51	2.98 ± 0.60
Mean duration of C (min)	9.57 ± 2.03	11.09 ± 2.90	12.36 ± 2.07
Duration to 1st C (min)	2.65 ± 0.58	4.63 ± 1.43**	2.01 ± 0.63
Duration of 1st C (min)	1.48 ± 0.74	0.86 ± 0.23	6.74 ± 4.14
Duration of E1 (h)	0.22 ± 0.08	0.43 ± 0.18	0.40 ± 0.10
Duration of E2 (h)	1.16 ± 0.45*	0.71 ± 0.43**	3.82 ± 0.76
Duration of G (h)	1.69 ± 0.57	1.37 ± 0.71	0.22 ± 0.15
Number of C	28.83 ± 6.50*	34.00 ± 5.62*	14.92 ± 2.80
Number of E1	2.25 ± 0.60	3.50 ± 1.03	2.92 ± 0.63
Number of E2	0.83 ± 0.30	1.25 ± 0.58	1.00 ± 0.28
Number of G	1.25 ± 0.41	0.33 ± 0.14	0.17 ± 0.11

Values are the means (±SE) of 12 biological replicates, and asterisks indicate significance of differences between the transgenic lines and the wild type (*P <* *0.05 and **P <* *0.01, Mann–Whitney *U* pairwise comparisons). np, no probing; C, stylet penetration; E1, salivation phase; E2, phloem sap ingestion; G, xylem ingestion.

### Effects of *TcCHS* overexpression on aphid performance under greenhouse conditions

Trials were carried out under more natural conditions in a semi‐open glasshouse to validate laboratory results. Neighbouring plots with two lines of *TcCHS* plants and wild type became naturally infested with cotton aphids during a 30‐d period. The mean numbers of aphids per leaf area were recorded at the end (Figure S2a‐d). The aphid population on wild type plants reached 4.51 aphids per cm^2^ leaf area on the abaxial side and 0.28 per cm^2^ on the adaxial side on day 30. In contrast, the number of aphids on transgenic plants remained 15‐ to 22‐fold lower, with only 0.21–0.32 aphids per cm^2^ leaf area (*P *<* *0.0001) (Table 3), exclusively on the abaxial side of the leaf (Figure S2e‐f). Overall, the mean inhibition ratio (IR) of aphids after 30 d of natural infestation was 93%–96% compared to wild type and followed the differences in expression of the *TcCHS* gene in lines CHS3 and CHS11 (Table 3).

**Table 3:** The aphid resistance of transgenic chrysanthemum plants and wild type

Plant lines	Mean aphids/leaf area (cm^2^)^a^		IR (%)^b^
	Abaxial surface**	Adaxial surface***	
Wild type	4.51 ± 0.98^A^	0.28 ± 0.07^A^	0
CHS3	0.32 ± 0.05^B^	0.00 ± 0.00^B^	93
CHS11	0.21 ± 0.05^B^	0.00 ± 0.00^B^	96

a Values are the means (±SE) of 10 biological replicates, and different superscripts indicate significantly different (**P <* *0.0001; ***P <* *0.05).

b Inhibition ratio relative to wild type plants at 30 Day. IR is given by (*N*_W_–*N*_T_)/*N*_W_ × 100, where *N*_W_ represent the mean of aphid number per leaf area on the wild type and *N*_T_ the mean of that on transgenic plant at 30 Day.

## Discussion

Aphids are serious agricultural pests in both greenhouses and fields, on both ornamental and food crops. Unlike the majority of insects, they engage in cyclical parthenogenesis which leads to rapid population growth and generation times as short as 5 d (Goggin, 2007). These traits create major challenges to the management of aphids. Breeding strategies in hexaploid chrysanthemum are almost entirely focused on conventional breeding by hybridization and vegetative propagation of elite lines (Deng *et al*., 2010). The drawback to this is that the combination of all desirable traits in one cultivar depends on luck rather than strategy. Recently, the use of genetic modification to enhance or introduce the biosynthesis of specific terpenoids in plants has become increasingly common and efficient for repelling aphids (Yu *et al*., 2014). Here, we introduced into chrysanthemum the *TcCHS* gene from pyrethrum involved in the formation of chrysanthemol, the first step in the synthesis of pyrethrins. The gene was placed under the control of the Rubisco small subunit promoter and resulted in chrysanthemol emission and accumulation of chrysanthemol glycoside. Aphids were repelled and deterred by both the volatile chrysanthemol and nonvolatile chrysanthemol glycoside. We propose that these two principles may target different organs in the aphid (exterior and interior) and thus may enhance the effectiveness of this gene for aphid control. The amount of stored chrysanthemol (1.1 mM) was approximately 1000‐fold higher than emitted per day per unit tissue as aglycon, suggesting a highly efficient glycosylation process. The *in vitro* bioactivity of the glycoside was lost at fourfold dilution suggesting that for dual effects with this aphid species 0.55 mM of the glycoside is required at minimum. Such high accumulation levels are not always attained in other plant species with other monoterpenes and will require a case by case evaluation (Yang *et al*., 2011).

The first *TcCHS* gene (accession number I13995 with an incorrectly spliced targeting signal) was cloned by Rivera and reported to only catalyse cyclopropane ring formation to give chrysanthemyl pyrophosphate (CPP) (Rivera *et al*., 2001). It yielded dwarfed phenotypes in tomato (Tang *et al*., 2012). The *TcCHS* gene (accession number JX913537) used in this study, had a normal plastid targeting signal and was shown in tobacco to catalyse the two step conversion of DMAPP to chrysanthemol via CPP leading to the emission of chrysanthemol and the accumulation of its derivative, but with dwarfed phenotypes as well (Yang *et al*., 2014). Biosynthesis of novel terpenoids in transgenic plants has been suggested to influence plant development by competing for isoprenoid precursor substrates or enzyme partners necessary for the formation of metabolites involved in primary metabolism, including carotenoids, chlorophyll and growth regulators (Aharoni *et al*., 2005). The *TcCHS* gene is normally exclusively expressed in trichome cells (Sultana *et al*., 2015). In our study, we used, like in tobacco, the strong rubisco small subunit promoter for efficient *TcCHS* overexpression in most aboveground cell types. This led to high emissions of the monoterpene chrysanthemol, but surprisingly without the negative effects on plant phenotype that were observed in tobacco. *CHS*‐like genes have been found in a range of Asteraceous plants including chrysanthemum (Liu *et al*., 2012). Possibly, this plant family carries adaptations to deal with the overexpression of this monoterpene synthase in ways that do not interfere with primary metabolism.

Examination of both the volatile and nonvolatile glycoside of chrysanthemol in choice assays with cotton aphids generated evidence that both molecules have the capacity to modify host plant selection and growth and reproduction when expressed in chrysanthemum. The aphid tissues contacted by these molecules will differ strongly as the glycoside will typically pass through the gustatory and digestive systems, whereas the volatile may contact exterior olfactory and respiratory systems. It leads to the question whether the molecular target of both molecules would be different as well. In that respect it may be relevant that the chrysanthemic acid moiety of pyrethroid esters is known to be required for the interaction with insect voltage‐gated sodium channels that are found throughout the insect body (Barr *et al*., 2010; Dong *et al*., 2014). Potentially, the unusually strong effects of this molecule actually relate to effects on sodium channels, which gives the insects a certain level of discomfort. Evidence of a direct effect of chrysanthemol on such channels has not yet been reported. However, future studies could test whether the strong repellent/deterrent activity of chrysanthemol or chrysanthemyl acetate and glycoside may be related to weak interactions with voltage‐gated sodium channels. Such studies could also establish that whether the glycoside is indeed itself bioactive or requires an enzymatic activation step to convert it into reactive aglycones by glycosidases from plants or insect saliva and gut enzymes (Pankoke *et al*., 2010).

We provided *in vitro* evidence that purified chrysanthemyl‐6‐O‐malonyl‐β‐D‐glucopyranoside, which has not been previously reported in the plant kingdom, is the main new compound different between the tested extracts and results in significant feeding deterrence at the concentrations found in our transgenic plant lines. However, a question is whether aphids actually contact this stored resource. We did not analyse it for the chrysanthemol glycoside, but in general, the default storage pathway of defence volatile isoprenoids and terpene glycosides appears to be the leaf mesophyll cells and vacuole (Fineschi and Loreto, 2012; Gabrys and Tjallingii, 2002; Ketudat Cairns and Esen, 2010). Aphids usually do probe vacuolar contents during intracellular punctures of mesophyll cells (Hewer *et al*., 2011), so that aphids may likely encounter and taste these compounds during stylet penetration before reaching phloem. Olfactory signals and initial probes are the major factors influencing initial host selection by aphids (Powell *et al*., 2006). When the composition of vacuolar sap contains deterrent components, this may lead to decisions to withdraw their stylets and continue penetrating and probing elsewhere (Hewer *et al*., 2011), and that may explain in part the extended no‐probing times and higher frequency of stylet penetration probes we observed in the EPG studies of the aphids (Gabrys and Tjallingii, 2002). If aphids do not encounter deterrents during probing of mesophyll cells, once a phloem sieve element is found, they will engage in prolonged periods of phloem ingestion as observed on wild type plants. This crucial feeding process was clearly severely shortened on both transgenics but the number of E2 events was not, confirming that the process of reaching the E2 phase appears to be affected most (Table 2).

In field trials in semi‐open greenhouses, two transgenic lines of chrysanthemum overexpressing *TcCHS* remained largely free of aphids during the entire 30‐d period in strong contrast to the wild type. The strong population reduction (>90%) of transgenic plants in field trials suggests that the gene may provide a powerful method to introduce resistance against cotton aphids into chrysanthemum. It will be interesting to evaluate effects of *TcCHS* products also on other pests and beneficial insects and to evaluate the application of the gene in other crops.

## Experimental procedures

### Plant materials

Chrysanthemum (*Chrysanthemum morifolium*) ‘1581’ is a cultivar highly susceptible to aphids. All plants were propagated vegetatively by cuttings in pots and were grown in a greenhouse under a 16/8‐h light/dark illumination at 25/20 °C day/night temperature.

### Aphid rearing

Cotton aphids (*Aphis gossypii*) were initially field‐collected and thereafter mass‐reared on chrysanthemum (*Chrysanthemum morifolium*) ‘ZC13’ in a greenhouse (25/20 °C day/night temperature, 16/8‐h light/dark illumination, relative humidity (RH) of 60%–70%). Before each bioassay, aphids at each stage were transferred to a petri dish and starved for 4 h. All laboratory bioassays were conducted in a climate room (20 °C, 16/8‐h light/dark, RH of 60%–70%).

### Generation of transgenic chrysanthemum

The full‐length *TcCHS* cDNA (GenBank: JX913537), including its native plastid targeting signal, was cloned into the modified binary vector pBINPLUS (Yang *et al*., 2014) under the control of the chrysanthemum RbcS1 promoter (Outchkourov *et al*., 2003). The expression vector (pBINPLUS‐CHS) was transferred into *Agrobacterium tumefaciens* strain AGL‐0 and used to transform chrysanthemum leaf explants (Mao *et al*., 2013). Two T_0_ transgenic lines, CHS3 and CHS11, were propagated and used for further chemical and biological analysis.

### Quantitative RT‐PCR

Expression of the *TcCHS* gene in leaves and ovaries of transgenic lines was analysed by real‐time qPCR as described (Zeng *et al*., 2016). The transcript levels of *TcCHS* were normalized relative to the *Actin* gene (GenBank: AB205087) from chrysanthemum. The forward (Fw) and reverse (Re) primers used were *CHS*‐Fw (5′‐CATCTTCTGGACCTCTTCAATGAG‐3′) and *CHS*‐Re (5′‐GTACTGAACAATCCGACGGTTAAG‐3′), and *Actin*‐Fw (5′‐TCCCGTCCTTCTTACTGAG‐3′) and *Actin*‐Re (5′‐CTGAGATAGCAACATACATAGC‐3′). Wild type plants were used as control.

### Phenotypic trait analysis

For chlorophyll and carotenoids measurement, 0.2 g of the third top leaf of 1‐month‐old plants was harvested and put into 25 mL extraction buffer with 80% acetone: 95% ethanol (2:1 v/v) for 48 h in the dark at 20 °C. The concentrations were measured as described (Lichtenthaler, 1987). All aboveground parts of 1‐month‐old plants were harvested for dry weight measurements. For leaf area analysis, six leaves were picked, equidistantly from top to bottom, from 3‐month‐old plants, and the mean area per leaf was calculated manually using gridlines. Three independent replicates per genotype were used in the analysis of variance (ANOVA) and Duncan's multiple range test (*P *<* *0.05).

### Volatile analysis by GC‐MS

To analyse the quantity and composition of intact plant emitted volatiles, a dynamic headspace trapping system was used in a growth chamber set‐up (20 ± 2 °C, 70% RH; L:D 12‐h photoperiod and 150–170 μmol photons/m^2^/s PPFD at the level of plants) as described (Ting *et al*., 2015). Each plant was placed inside half‐closed 1‐L jars with inlet and outlet stainless steel cartridges filled with 200 mg Tenax TA (20/35 mesh; Grace‐Alltech, Deerfield, MI). Plant volatiles were collected in the outlet cartridge for 4 h. Shoot fresh weight was determined immediately after trapping.

Headspace samples were analysed with an Agilent GC 7890B series connected to an Agilent 7200 series Q‐TOF mass spectrometer. After dry purging, the Tenax cartridges were desorbed with a helium flow (30 mL/min) at 240 °C for 3 min (Model Ultra Markes Llantrisant, UK). Analytes were focused at 0 °C on an online electronically cooled sorbent trap (Unity, Markes). Subsequently, the sorbent trap was quickly heated to 260 °C for 3 min and the analytes were injected on the analytical column (DB‐5MS, 30 m × 0.25 mm ID, 1.0 μm film thickness, Agilent) with a split flow of 40 to 100 mL/min, depending on the injected samples. The GC temperature program was 40 °C for 2 min, rising to 280 °C at 10 °C/min and 280 °C for 4 min. The electron impact ionization was 70 eV. Mass scanning was performed from 50 to 400 m/z with 5 scans/s. The temperature of the ion source and transfer line was 230 and 280 °C, respectively.

Results were qualitatively and quantitatively analysed using MassHunter Acquisition Data B.07.00. Constituents were identified with the NIST library (version 2.2, 2014) based on retention time and mass spectrum. Chrysanthemol was identified with an authentic standard (Sigma, Saint Louis, MO). The standard of chrysanthemyl acetate was prepared by acetylation of chrysanthemol as described (Mojtahedi and Samadian, 2013). The chrysanthemol emission from transgenic plants was quantified based on calibration curves with the authentic standard.

### Nonvolatile analysis by LC‐MS

Leaves (0.2 g) from the top of young cuttings of wild type and T_0_ chrysanthemum lines were used for nonvolatile analysis according to a protocol for untargeted metabolomics of plant tissues as previously described in detail (Yang *et al*., 2011). LC‐MS analysis was performed using an Agilent 1260 HPLC connected to an Agilent 6520 Accurate‐Mass Q‐TOF LC/MS (Agilent) operating in negative ionization. The column used was a ZORBAX Eclipse plus C18 (1.8 μm) LC column (2.1 × 100 mm). The gradient started at 5% B (acetonitrile:formic acid [1000:1, v/v]) and increased linearly to 35% in 50 min at 0.2 mL/min. The injection volume was 5 μL.

### GC‐MS and LC‐MS data processing

GC‐MS and LC‐MS data were acquired using MassHunter (Agilent). The data sets were then processed simultaneously using the dedicated MetAlign metabolomics software (http://www.metAlign.nl) for automated baseline correction and subsequent spectral data alignment (De Vos *et al*., 2007). The processing parameters of MetAlign for GC‐MS data were set to analyse scan numbers 1 to 7381 with a maximum amplitude of 1 × 10^8^. The parameters for LC‐MS data were set to analyse scan numbers 1 to 9100 with a maximum amplitude of 1 × 10^6^.

After MetAlign analysis, mass signal clustering was conducted by MSClust (Tikunov *et al*., 2012). For each extracted cluster, the average mass intensity was used to calculate the intensity ratio of transgenic to control plants. Significant differences between plants were then determined by the Student's *t*‐test. Masses with a significant (*P *<* *0.05) and more than fivefold intensity change were verified manually in the original chromatograms.

### Glycosidase treatment

To characterize glycosylated chrysanthemol, 0.2 g leaves from wild type and transgenic lines were ground in liquid nitrogen and extracted with 1 mL citrate phosphate buffer (200 mm Na_2_HPO_4_, 220 mm citric acid, pH 5.0). 200 μL of β‐glycosidase (Sigma) was added. The mixture was overlaid by 500 μL pentane and incubated at 37 °C for 6 h and subsequently extracted by 500 μL ethyl acetate. Extracts were dehydrated using Na_2_SO_4_ and then analysed using GC‐MS as described (Yang *et al*., 2013).

### Purification and identification of chrysanthemyl‐6‐O‐malonyl‐β‐D‐glucopyranoside

Plants extracts were prepared in ethanol as described above, and then, the extracts were prepurified using a Bond Elut LRC‐C18 500 mg column (Agilent). The new compound in transgenic plants later identified as chrysanthemyl‐6‐O‐malonyl‐β‐D‐glucopyranoside was further purified with a preparative LC‐MS system as previously described (Yang *et al*., 2011). The gradient started at 80% B (acetonitrile:formic acid [1000:1, v/v]) and increased linearly to 100% in 30 min. The fraction containing the molecular ion of the compound ([M‐H]^−^ = 803.368) was manually collected. Subsequently, the fractions purified from 10 g leaves were freeze‐dried and redissolved in deuterated dimethyl sulfoxide (DMSO) to be analysed by NMR. NMR procedures were described by Yang *et al*. (2011). The concentration of chrysanthemyl‐6‐O‐malonyl‐β‐D‐glucopyranoside in transgenic plants was quantified based on calibration curves with the pure compound after NMR measurement.

### Aphid performance

Six wild type and transgenic plants were clonally propagated from rooted shoot cuttings for a nonchoice assay to test aphid performance. Ten neonate aphid nymphs were introduced into a clip cage holding a leaf. One leaf per plant was tested. Plants were watered every 2 days. The total number of aphids was recorded after a 12‐day period on six replicates. Significant differences in survival were analysed using variance (ANOVA) and Duncan's multiple range tests (*P *<* *0.05).

### Aphid olfactory dual‐choice assay on detached leaves

The aphid behavioural response based on olfactory cues was tested using a dual‐choice assay with detached leaves from wild type or transgenic plants as described (Yang *et al*., 2013). Aphids (from the stock rearing) that chose a leaf within five minutes were scored. The test was replicated with ten pairs of leaves, each with ten individual aphids. Two‐tailed Wilcoxon signed rank test (*P *<* *0.05) was used to analyse the significant preference of aphids based on olfactory cues. The choice between filter paper discs (1 cm diameter) applied with 10 μL 10% chrysanthemol standard dissolved in paraffin oil, and 10 μL paraffin oil was used as positive control.

### Aphid dual‐choice assay on intact plants

Dual‐choice assays on intact plants were conducted to detect aphid preference. Young plants of 10 cm (WT and line CHS3, CHS11), separated by a water barrier, were connected by a paper bridge (2 × 4 cm). Ten moving wingless aphids of mixed age (from the stock rearing) were released at the centre of the bridge. The number of aphids on each plant was counted at 0.25, 1, 3, 6 and 16 h after release. Six replicates were set up for each assay. Two‐tailed Wilcoxon signed rank test (*P *<* *0.05) was used to analyse the significant preference of aphids on transgenic or wild type plants.

### Aphid alarm response assay

The alarm response assay of Beale *et al*. (2006) was used, with some modifications, to test the effects of chrysanthemol on settling aphids. Rooted plantlets with three young leaves of chrysanthemum ‘ZC13’ were enclosed in a 1 L vessel and inoculated with ten fourth‐instar aphids (from the stock rearing) on each plant. After 1 h stable feeding, 500 ng pure chrysanthemol diluted in 100 μL paraffin oil was sprayed on the wall of the vessel and volatilized. The aphid behaviour was observed, and the number of aphids moving after 5 min and 10 min was recorded. The amount of chrysanthemol was in the range of that emitted into the headspace during 1 h by a whole transgenic line 3 plant (3.4–12.3 ng/g, ca. height 50 cm, weight 40 g). Paraffin oil was used as solvent control. Each assay was performed with twenty replicates. Significant differences between treatments were analysed using variance (ANOVA) and Duncan's multiple range tests (*P *<* *0.05).

### Aphid dual‐choice assay with leaf nonvolatile contents and pure glycoside

Methanol extracts from young chrysanthemum leaves and dual‐choice assay set‐up were prepared as previously described (Yang *et al*., 2011). The methanol extracts were then freeze‐dried, and the residue equivalent to 1 g leaves redissolved in 1 mL 25% sugar water to achieve the same concentration as in leaves. Two droplets (100 μL) of either transgenic or wild type sugar water extract were added on the top of parafilm on the dish. Each dish was inoculated with ten aphids which were starved overnight. Preference of aphids was determined by scoring the number of aphids underneath the droplets every 15 min. For data analysis, averages of five replicates of two dual‐choice plates were used for each three‐hour time interval.

The dual‐choice assay with pure chrysanthemol glycoside was carried out using automated video tracking system as described (Thoen *et al*., 2016). We dissolved the chrysanthemol glycoside in 25% sugar water at the same concentration (434 μg/mL, 1.08 mm) which in transgenic chrysanthemum and further prepared a series of dilutions (217, 108.5, 54.25, 0 μg/mL). 25% sugar water was used as solvent control. Eight arenas per dilution were monitored during eight consecutive hours. The assay was replicated seven times. The duration under sample and control solutions relative to total detected time were recorded. Two‐tailed Wilcoxon signed rank test (*P *<* *0.05) was used to analyse the significant preference.

### Aphid probing behaviour

The feeding behaviour of adult apterous aphids was monitored using a Giga‐4‐DC‐EPG system (Tjallingii, Wageningen, the Netherlands). The feeding activities of aphids were monitored continuously for 8 h on the third leaf of chrysanthemum plants in a faraday cage in the laboratory (20 °C, RH of 60%–70%). EPG recordings were made of 12 aphids per genotype. Data were recorded and analysed with the Stylet+a software, and waveforms were interpreted according to Tjallingii (1986). Data were subjected to an analysis of variance followed by the Mann–Whitney *U*‐test.

### Greenhouse trial with natural aphid infestation

To determine the resistance of transgenic chrysanthemum plants, plants grown under the same conditions were used for natural infestation by aphids for 30 d during the spring aphid season. Six transgenic lines CHS3, six CHS11 and six control lines were each arranged in pots on the floor in three separate 3 m long × 2 m wide rectangular plots so that the volatiles emitted from each line had least effect on neighbouring lines. All plants were grown in an open greenhouse. Plants were given normal water management and no pest control. Thirty days after infestation, six leaves per plant were picked equidistantly, from top to bottom, and the numbers of aphids on the abaxial and adaxial surfaces, and the leaf area, were recorded. Resistance was quantified by the mean number of aphids per leaf area and an inhibition ratio (IR) relative to control. IR is given by [(*N*_W_–*N*_T_)/*N*_W_] × 100, where *N*_W_ represents the mean aphid number per leaf area on the wild type and *N*_T_ the mean of that on transgenic plants at day 30. The difference between means of the data was analysed using ANOVA, with *P *<* *0.05 considered to indicate significant differences.

## Supporting information


**Table S1** Volatile metabolites with a >5‐fold intensity change between transgenic lines and wild type plants.
**Table S2** Non‐volatile metabolites with a >5‐fold intensity difference between transgenic lines and wild type plants.
**Table S3** NMR data for chrysanthemyl‐6‐O‐malonyl‐β‐D‐glucopyranoside in acetonitrile D3 (300 K).
**Figure S1** GC‐MS chromatograms of leaf extracts after deglycosylation with β‐glycosidase of (a) wild type and (b) *TcCHS* transgenic plants. (c) GC‐MS chromatograms of leaf extracts without β‐glycosidase of *TcCHS* transgenic plants. (d) GC‐MS chromatograms of an authentic standard of chrysanthemol.
**Figure S2** Field trial by natural aphid infestation on wild type and transgenic chrysanthemum plants. aphid proliferation on 3‐month‐old wild type (a) and transgenic plant (d) at 30 d. The dotted line represents the position to zoom‐in. (b) zoom‐in picture of wild type plant. (c) zoom‐in picture of transgenic plant. Aphid proliferation on leaves of wild type (e) and transgenic plant (f) at 30 d. Bar 1 cm.
