# Supplementary material for: Modification of chrysanthemum odour and taste with chrysanthemol synthase induces strong dual resistance against cotton aphids
Source: Plant Biotechnol J. 2018 Jul 11;16(8):1434–45. doi: 10.1111/pbi.12885 (PMC6041446; doi:10.1111/pbi.12885)
Supplement: Supplementary file 1 — Table S1 Volatile metabolites with a >5‐fold intensity change between transgenic lines and wild type plants. Table S2 Non‐volatile metabolites with a >5‐fold intensity difference between transgenic lines and wild type plants. Table S3 NMR data for chrysanthemyl‐6‐O‐malonyl‐β‐D‐glucopyranoside in acetonitrile D3 (300 K). Figure S1 GC‐MS chromatograms of leaf extracts after deglycosylation with β‐glycosidase of (a) wild type and (b) TcCHS transgenic plants. (c) GC‐MS chromatograms of leaf extracts without β‐glycosidase of TcCHS transgenic plants. (d) GC‐MS chromatograms of an authentic standard of chrysanthemol. Figure S2 Field trial by natural aphid infestation on wild type and transgenic chrysanthemum plants. aphid proliferation on 3‐month‐old wild type (a) and transgenic plant (d) at 30 d. The dotted line represents the position to zoom‐in. (b) zoom‐in picture of wild type plant. (c) zoom‐in picture of transgenic plant. Aphid proliferation on leaves of wild type (e) and transgenic plant (f) at 30 d. Bar 1 cm. [file PBI-16-1434-s001.doc]

**Table S1** Volatile metabolites with a >5-fold intensity change between transgenic lines and wild-type plants

| Ret (min) | Av intensity (CHS3) | Av intensity (CHS11) | Av intensity (WT) | Ratio (CHS3/WT) | Ratio (CHS11/WT) | Ratio (CHS11/CHS3) | S/N (CHS3) | S/N (CHS11) | S/N (WT) | Mass Fragments |
| --- | --- | --- | --- | --- | --- | --- | --- | --- | --- | --- |
| 14.70 | 36059 | 54580 | 1136 | 31.73 | 48.03 | 1.51 | 36.1 | 54.6 | 1.1 | 123, 136 |
| 14.92^a^ | 8154583 | 9623116 | 1085 | 7514.69 | 8867.99 | 1.18 | 8154.6 | 9623.1 | 1.1 | 123, 81, 79, 67, 93 |
| 14.95 | 1048 | 2997 | 23899 | 0.04 | 0.13 | 2.86 | 1.0 | 3.0 | 23.9 | 105, 123, 95, 83 |
| 15.11 | 20197 | 24955 | 2685 | 7.52 | 9.29 | 1.23 | 20.2 | 25.0 | 2.7 | 77, 111 |
| 16.71^b^ | 168107 | 204867 | 1065 | 157.79 | 192.29 | 1.22 | 168.1 | 204.9 | 1.1 | 121, 123, 91, 77 |

Only differences are listed with > 5-fold differences that are significant by the Student’s t test (*P* < 0.05).

Ret (min), retention time, in minutes; Av, average; WT, wild-type; Ratio (CHS3/WT), ratio of mass signal between transgenic line (CHS3) and wild-tpye (WT); Ratio (CHS11/WT), ratio of mass signal between transgenic line (CHS11) and wild-tpye (WT); Ratio (CHS11/CHS3), ratio of mass signal between transgenic lines CHS11 and CHS3; S/N, ratio of mass signal to noise.

^a^ Retention time of trans-chrysanthemol.

^b^ Retention time of trans-chrysanthemyl acetate.

**Table S2** Non-volatile metabolites with a > 5-fold intensity difference between transgenic lines and wild-type plants

| Ret (min) | Av intensity (CHS3) | Av intensity (CHS11) | Av intensity (WT) | Ratio (CHS3/WT) | Ratio (CHS11/WT) | Ratio (CHS11/CHS3) | S/N (CHS3) | S/N (CHS11) | S/N (WT) | Mass Fragments |
| --- | --- | --- | --- | --- | --- | --- | --- | --- | --- | --- |
| 11.31 | 8058 | 8019 | 54214 | 0.15 | 0.15 | 1.00 | 1.0 | 1.0 | 6.8 | 377, 445 |
| 14.05 | 8110 | 8109 | 83448 | 0.10 | 0.10 | 1.00 | 1.0 | 1.0 | 10.4 | 311, 275, 343 |
| 16.69 | 8093 | 8079 | 56649 | 0.14 | 0.14 | 1.00 | 1.0 | 1.0 | 7.1 | 321, 775 |
| 17.26 | 568776 | 184882 | 8175 | 69.58 | 22.62 | 0.33 | 71.1 | 23.1 | 1 | 387, 388, 775 |
| 20.20 | 116475 | 100828 | 8157 | 14.28 | 12.36 | 0.87 | 14.6 | 12.6 | 1 | 377, 378 |
| 20.91 | 195150 | 219570 | 8079 | 24.15 | 27.18 | 1.13 | 24.4 | 27.4 | 1 | 377, 378 |
| 21.58 | 168076 | 114045 | 8174 | 20.56 | 13.95 | 0.68 | 21.0 | 14.3 | 1 | 509, 577, 463 |
| 24.22 | 9325 | 12479 | 66262 | 0.14 | 0.19 | 1.34 | 1.2 | 1.6 | 8.3 | 347, 383, 385 |
| 26.16 | 172410 | 153273 | 8071 | 21.36 | 18.99 | 0.89 | 21.6 | 19.2 | 1 | 373, 374, 419 |
| 27.02 | 142511 | 243513 | 8055 | 17.69 | 30.23 | 1.71 | 17.8 | 30.4 | 1 | 853, 836, 419 |
| 29.02 | 57225 | 84611 | 8161 | 7.01 | 10.37 | 1.48 | 7.2 | 10.6 | 1 | 373, 374 |
| 32.75 | 76081 | 107532 | 13166 | 5.78 | 8.17 | 1.41 | 9.5 | 13.4 | 1.6 | 415, 919 |
| 38.04 | 108704 | 128554 | 8145 | 13.35 | 15.78 | 1.18 | 13.6 | 16.1 | 1 | 493, 494 |
| 38.88 | 247181 | 150655 | 8007 | 30.87 | 18.81 | 0.61 | 30.9 | 18.8 | 1 | 447, 561, 483 |
| 40.73 | 53719 | 53191 | 8078 | 6.65 | 6.58 | 0.99 | 6.7 | 6.6 | 1 | 493, 494 |
| 46.88^a^ | 1750844 | 2092640 | 8173 | 214.22 | 256.04 | 1.20 | 218.9 | 261.6 | 1 | 803, 401, 357 |

Only differences are listed with > 5-fold differences that are significant by the Student’s t test (*P* < 0.05).

Ret (min), retention time, in minutes; Av, average; WT, wild-type; Ratio (CHS3/WT), ratio of mass signal between transgenic line (CHS3) and wild-type (WT); Ratio (CHS11/WT), ratio of mass signal between transgenic line (CHS11) and wild-type (WT); Ratio (CHS11/CHS3), ratio of mass signal between transgenic lines CHS11 and CHS3; S/N, ratio of mass signal to noise.

^a^ Retention time of chrysanthemyl-6-O-malonyl-β-D-glucopyranoside.

**Table S3** NMR data for chrysanthemyl-6-O-malonyl-β-D-glucopyranoside in acetonitrile D3 (300 K).

| Position | ^δ13^C | ^δattached^H |
| --- | --- | --- |
| chrysanthemyl |  |  |
| 1 | 70.4 | 3.90/3.48 |
| 2 | 33.1 | 0.79 |
| 3 | 22.6 | - |
| 4 | 29.0 | 1.12 |
| 5 | 124.5 | 4.90 |
| 6 | 133.7 | - |
| 7 | 25.6 | 1.69 |
| 8 | 18.3 | 1.66 |
| 9 | 22.7 | 1.02 |
| 10 | 21.6 | 1.10 |
| glucose |  |  |
| 1’ | 103.1 | 4.22 |
| 2’ | 74.6 | 3.08 |
| 3’ | 77.4 | 3.28 |
| 4’ | 70.9 | 3.28 |
| 5’ | 74.4 | 3.41 |
| 6’ | 65.2 | 4.34/4.26 |
| malonyl |  |  |
| 1’’ | 167.9 | - |
| 2’’ | 41.6 | 3.37/3.37 |
| 3’’ | 168.0 | - |


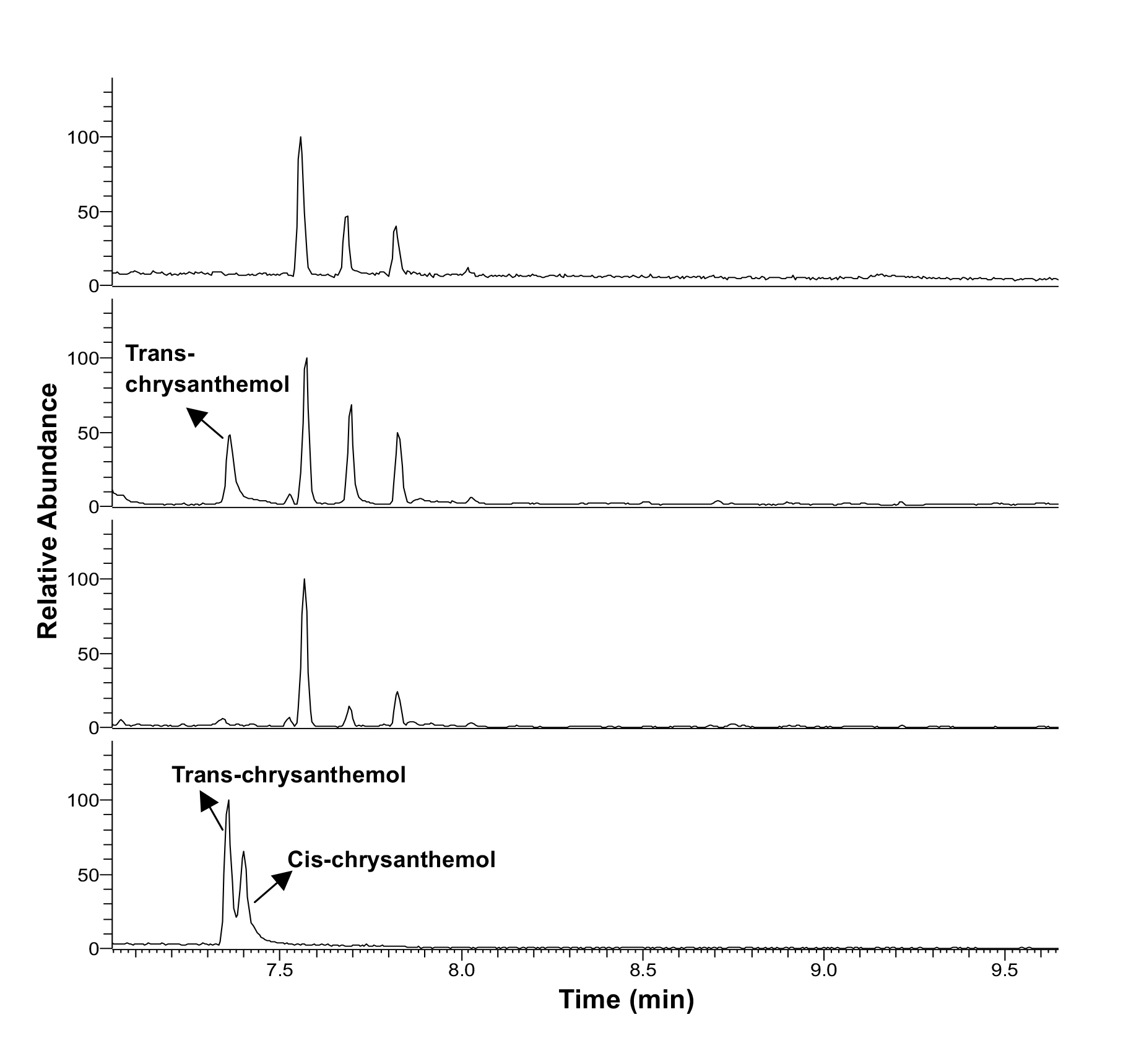


(a)

(b)

(c)

(d)

**Figure S1** GC-MS chromatograms of leaf-extracts after deglycosylation with β-glycosidase of (a) wild-type and (b) *TcCHS* transgenic plants. (c) GC-MS chromatograms of leaf-extracts without β-glycosidase of *TcCHS* transgenic plants. (d) GC-MS chromatograms of an authentic standard of chrysanthemol.


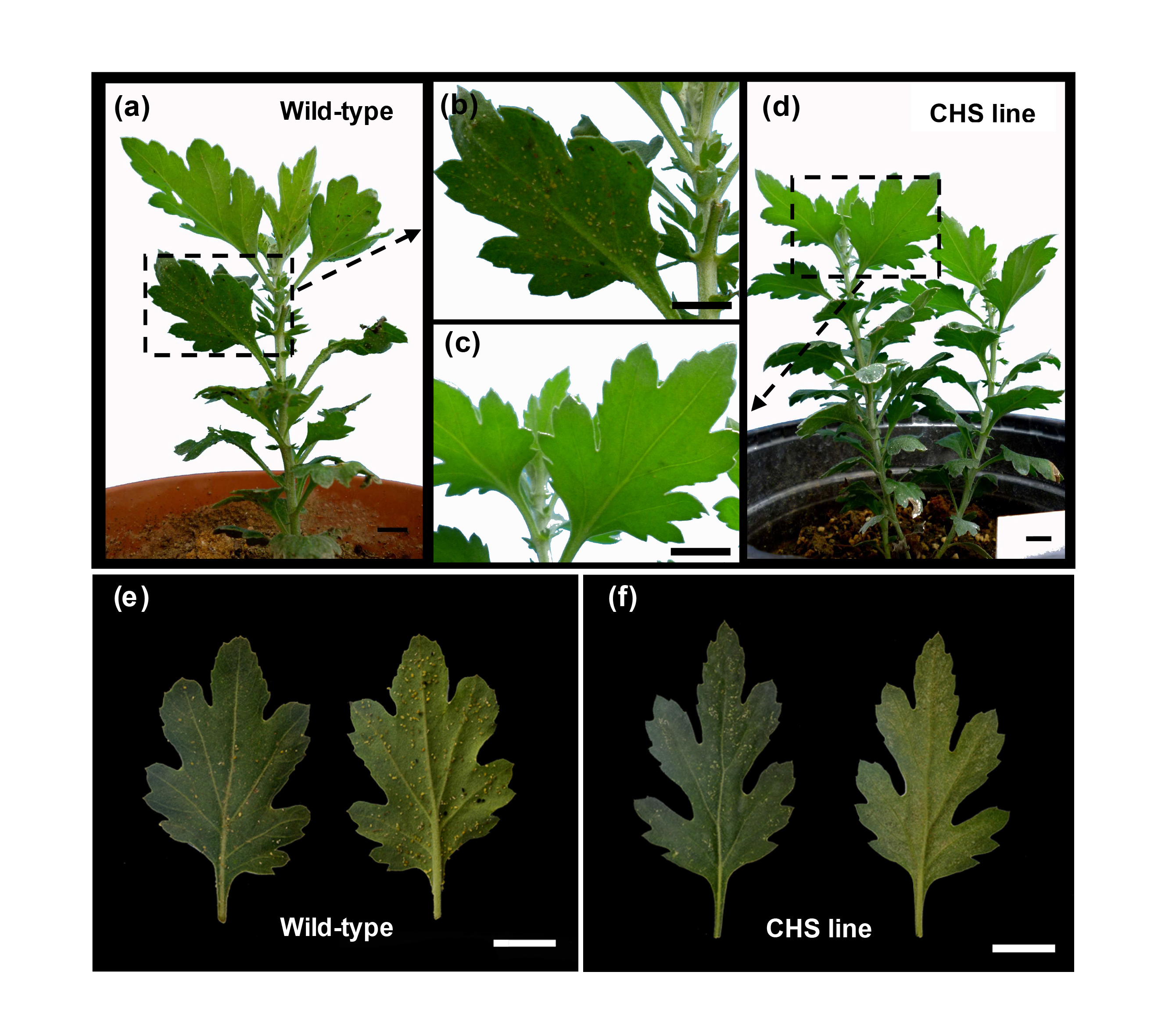


**Figure S2** Field trial by natural aphid infestation on wild-type and transgenic chrysanthemum plants. aphid proliferation on three-month-old wild-type (a) and transgenic plant (d) at 30 d. The dotted line represents the position to zoom-in. (b) zoom-in picture of wild-type plant. (c) zoom-in picture of transgenic plant. Aphid proliferation on leaves of wild-type (e) and transgenic plant (f) at 30 d. Bar 1cm.
